# Pyruvate dehydrogenase B regulates myogenic differentiation via the FoxP1–Arih2 axis

**DOI:** 10.1002/jcsm.13166

**Published:** 2022-12-23

**Authors:** Xuan Jiang, Siyu Ji, Fenglai Yuan, Tushuai Li, Siyuan Cui, Wei Wang, Xianlong Ye, Rong Wang, Yongquan Chen, Shenglong Zhu

**Affiliations:** ^1^ Wuxi School of Medicine Jiangnan University Wuxi China; ^2^ School of Food Science and Technology Jiangnan University Wuxi China; ^3^ Institute of Integrated Traditional Chinese and Western Medicine Affiliated Hospital of Jiangnan University Wuxi China; ^4^ Wuxi No. 2 People's Hospital Wuxi China; ^5^ Ganjiang Chinese Medicine Innovation Center Nanchang China; ^6^ School of Translational Medicine Jiangnan University Wuxi China

**Keywords:** ageing, myogenesis, PDHB, sarcopenia

## Abstract

**Background:**

Sarcopenia, the age‐related decline in skeletal muscle mass and function, diminishes life quality in elderly people. Improving the capacity of skeletal muscle differentiation is expected to counteract sarcopenia. However, the mechanisms underlying skeletal muscle differentiation are complex, and effective therapeutic targets are largely unknown.

**Methods:**

The human Gene Expression Omnibus database, aged mice and primary skeletal muscle cells were used to assess the expression level of pyruvate dehydrogenase B (PDHB) in human and mouse aged state. d‐Galactose (d‐gal)‐induced sarcopenia mouse model and two classic cell models (C2C12 and HSkMC) were used to assess the myogenic effect of PDHB and the underlying mechanisms via immunocytochemistry, western blotting, quantitative real‐time polymerase chain reaction, RNA interference or overexpression, dual‐luciferase reporter assay, RNA sequencing and untargeted metabolomics.

**Results:**

We identified that a novel target PDHB promoted myogenic differentiation. PDHB expression decreased in aged mouse muscle relative to the young state (−50% of mRNA level, *P* < 0.01) and increased during mouse and primary human muscle cell differentiation (+3.97‐fold, *P* < 0.001 and +3.79‐fold, *P* < 0.001). Knockdown or overexpression of PDHB modulated the expression of genes related to muscle differentiation, namely, myogenic factor 5 (*Myf5*) (−46%, *P* < 0.01 and −27%, *P* < 0.05; +1.8‐fold, *P* < 0.01), myogenic differentiation (*MyoD*) (−55%, *P* < 0.001 and −34%, *P* < 0.01; +2.27‐fold, *P* < 0.001), myogenin (*MyoG*) (−60%, *P* < 0.001 and −70%, *P* < 0.001; +5.46‐fold, *P* < 0.001) and myosin heavy chain (*MyHC*) (−70%, *P* < 0.001 and −69%, *P* < 0.001; +3.44‐fold, *P* < 0.001) in both C2C12 cells and HSkMC. Metabolomic and transcriptomic analyses revealed that PDHB knockdown suppressed pyruvate metabolism (*P* < 0.001) and up‐regulated ariadne RBR E3 ubiquitin protein ligase 2 (Arih2) (+7.23‐fold, *P* < 0.001) in cellular catabolic pathways. The role of forkhead box P1 (FoxP1) (+4.18‐fold, *P* < 0.001)‐mediated Arih2 transcription was the key downstream regulator of PDHB in muscle differentiation. PDHB overexpression improved d‐gal‐induced muscle atrophy in mice, which was characterized by significant increases in grip strength, muscle mass and mean muscle cross‐sectional area (1.19‐fold to 1.5‐fold, *P* < 0.01, *P* < 0.05 and *P* < 0.001).

**Conclusions:**

The comprehensive results show that PDHB plays a sarcoprotective role by suppressing the FoxP1–Arih2 axis and may serve as a therapeutic target in sarcopenia.

## Introduction

Skeletal muscle is the most abundant tissue in the body and is critical for a range of biological processes, such as metabolism, respiration and motion. Myogenesis is critical for systemic homeostasis and for the physiological and metabolic functions of the skeletal muscle.^1^, ^2^ Decreased myogenic differentiation capacity of skeletal muscle is causally linked to the development of sarcopenia (ageing‐induced loss of skeletal muscle mass and strength).^3^, ^4^ As the skeletal muscle ages, myogenesis gradually deteriorates, contributing to muscle mass loss.^5^ Impaired muscle function due to muscle atrophy is one of the important causes of the increased mortality in elderly people versus young people.^6^, ^7^ Therefore, preventing sarcopenia by enhancing muscle mass and function is expected to prolong the healthy life span. Skeletal myogenesis is a highly coordinated and intricate process that involves satellite‐cell activation, myogenic cell proliferation, cell‐cycle exit and fusion of myogenic cells into multinucleated muscle fibres.^8^ Intrinsically, myogenic regulatory factors (MRFs), such as myogenic factor 5 (Myf5), myogenic differentiation (MyoD) and myogenin (MyoG), are key transcription factors (TFs) that play vital roles in transcriptional regulation during myogenesis.^9^ However, little is known about the precise mechanisms and key players of sarcopenia. There is no approved anti‐sarcopenic medicine, and most of the currently developing therapeutic drugs are discontinued due to ineffective or side effects.^10^ Therefore, novel drug targets are urgently needed for the effective treatment of sarcopenia.

Current studies indicate that pyruvate may be used as a novel senolytic, and growing evidence has demonstrated that pyruvate metabolism is critical for the pathophysiology of skeletal muscle ageing.^11^ In addition, pyruvate dehydrogenase B (PDHB), a mitochondrial enzyme, catalyzes the conversion of glucose‐derived pyruvate to acetyl‐CoA and thus regulates the critical link between glycolysis and the citric acid cycle.^12^ Moreover, PDHB also plays an important role in oxidative phosphorylation, and the typical clinical presentation of PDHB deficiency includes encephalopathy, hypotonia, respiratory difficulties and lactic acidosis.^13^, ^14^ Previous research has established a positive correlation between PDHB and intramuscular fat concentration.^15^ Furthermore, a study has suggested a transcriptional regulatory relationship between MyoG and PDHB in skeletal muscle cells.^16^ Nonetheless, the biological function and action mechanism of PDHB in myogenesis are still unidentified.

In this study, we uncovered a hitherto unknown function of the PDHB‐modulated FoxP1–Arih2 axis in skeletal muscle regeneration and ageing, paving the way for the development of innovative therapeutic strategies against muscular ageing and degenerative diseases.

## Materials and methods

### Gene Expression Omnibus analysis

We assessed the microarray data of skeletal muscle biopsies from 36 subjects in the Gene Expression Omnibus (GEO) database (Accession Number GSE25941) by using the GEO2R method (https://www.ncbi.nlm.nih.gov/geo/info/geo2r.html). The young (25 ± 1 years old) participants included 7 men and 8 women (*n* = 15); the old (78 ± 1 years old) participants included 10 men and 11 women (*n* = 21). The biopsies were obtained from the vastus lateralis at the basal state.

### Animal studies

All the experiments were performed in accordance with the guidelines of the Animal Experimentation Ethics Committee of Jiangnan University (JN No. 20220615c0321229 [275] and JN No. 20220615c0401229 [276]). C57BL/6 mice were purchased from Jicui Yaokang Biological Technology Co. (Nanjing, China). For the ageing experiments, 3‐ and 22‐month‐old male mice (*n* = 4, per group) were used. Mice were maintained at 24 ± 2°C with 30% relative humidity on a 12/12 h light/dark cycle and provided with standard pellet food and water ad libitum. To induce skeletal muscle regeneration, 50 μL cardiotoxin (CTX, 10 μM, Sigma, USA, diluted in PBS) and 50 μL sterile PBS (control) were injected into the left and right tibial anterior (TA) muscle per anaesthetized mouse, respectively. The mice were sacrificed 3, 7 and 15 days after injection, and the TA muscles were then immediately harvested and snap frozen for RNA and protein analyses. For the d‐galactose (d‐gal)‐induced sarcopenia mice model experiments, eighteen 12‐week‐old male C57BL/6J mice were randomly divided into three groups as follows: (1) The control group was treated with saline for 12 weeks (control, *n* = 6) and also treated with lentivirus vehicle for 4 weeks starting at Week 8; (2) the d‐gal group was treated with d‐gal (150 mg/kg) for 12 weeks (d‐gal, *n* = 6) and also treated with lentivirus vehicle for 4 weeks starting at Week 8; and (3) the d‐gal plus mPDHB group was treated with d‐gal (150 mg/kg) for 12 weeks (d‐gal + mPDHB, *n* = 6) and also treated with PDHB‐overexpressing lentivirus for 4 weeks starting at Week 8. d‐Gal was administered intraperitoneally every other day, and lentivirus (1 × 10^8^ IU/mL) was injected into the bilateral gastrocnemius muscles of mice (three sites per side, 10 μL per site) twice a week. d‐Gal was ordered from Source Leaf Biotech (Shanghai, China). Overexpression lentiviruses for mouse PDHB and lentivirus vehicle were obtained from Transheep Bio (Shanghai, China). Grip strength was assessed using YLS‐13A Grip Strength Meter (Zhenghua Biological Instrument Co., China). Contents of lactic acid and calcium in gastrocnemius muscle were determined using lactate assay kit and calcium ion detection kit, respectively (A019‐1‐1 and C004‐2‐1, Jiancheng, Nanjing, China).

### Haematoxylin and eosin staining

For histological analyses, isolated muscle tissues were pre‐washed with normal saline and fixed for at least 24 h in 4% paraformaldehyde. After immersion fixation and dehydration embedding, paraffin sections (5 μm) of the muscles were prepared on a microtome (Leica RM2235). Paraffin sections of skeletal muscles were stained with haematoxylin and eosin (H&E) for evaluation of the morphology and regeneration of TA muscle and gastrocnemius muscle. The H&E staining images of muscle sections were recorded using Pannoramic MIDI system (3DHISTECH Ltd.). Image‐Pro Plus software was used to analyse the mean muscle fibre cross‐sectional area (CSA).

### Cell culture

C2C12 mouse myoblasts were provided by the Chinese Academy of Sciences Cell Bank (Shanghai, China). Primary human skeletal muscle cell line (HSkMC) (bio‐089926) was obtained from Biobw (Beijing, China). The cells were cultured in DMEM with 10% foetal bovine serum and 1% penicillin/streptomycin (PWL062, Meilunbio, China) at 37°C with 5% CO_2_. After reaching ~80–90% confluency, myoblasts were differentiated in DMEM with 2% horse serum (SH30074.03, HyClone, USA) for 3 or 5 days. A lactate assay kit (A019‐1‐1, Jiancheng, Nanjing, China) was used to determine the lactate levels in culture supernatants and cells. Primary muscle cells were isolated from gastrocnemius muscles of young and ageing C57BL/6J mice as described in literature.^17^

### RNA interference and overexpression

The negative control (si‐NC) for in vitro RNA interference (RNAi) was obtained from GenePharma (Shanghai, China). All the specific siRNA sequences used for RNAi are listed in *Table* *S1*. The coding sequence of the mouse *Pdhb* gene was cloned into the pcDNA3.1 expression vector to generate the PDHB expression vector (Genewiz). C2C12 cells and HSkMC were seeded into 6‐ or 12‐well plates. After 12 h, the cells were transfected with siRNAs or expression plasmids by using jetPRIME transfection reagent (114‐15, Polyplus). After 6 h, the cells were washed twice with PBS and incubated in the differentiation medium (DM) for 3 or 5 days to induce myotube formation.

### Immunofluorescence staining

For cytological staining, C2C12 cells and HSkMC were first fixed in 4% PFA for 20 min. Then, they were permeabilized in 0.3% Triton X‐100 for 10 min and blocked with 3% bovine serum albumin for 1 h. Afterward, the cells were immunostained with a specific antibody against MyHC (MF‐20, 1:50, DSHB), and DAPI (MA0128, Meilunbio, China) was used to counterstain the nuclei. Immunostained samples were photographed using inverted fluorescence microscopy (Ti‐U, Nikon). The number and area of MyHC+ myotubes were determined using five randomly selected high‐power fields (100×) per group. Fusion index (%) corresponds to the number of nuclei within myotubes, divided by the total number of nuclei counted.

### RNA extraction and quantitative real‐time polymerase chain reaction

Total RNA was extracted from C2C12, HSkMC and the skeletal muscle by using an RNA extraction kit (K101, JN.BIOTOOLS, Wuxi, China). Reverse transcription assay was performed in a 20 μL reaction volume with 1 μg total RNA by using the PrimeScript RT Reagent Kit with gDNA Eraser (Perfect Real Time) (RR047A, Takara). The SYBR Green Master Mix (11201ES03, Yeasen, Shanghai, China) was used for quantitative real‐time polymerase chain reaction (qRT‐PCR), and the reactions were conducted in a CFX96™ Real‐Time Detection System (Bio‐Rad). β‐Actin was used for data normalization through the 2^−ΔΔCt^ method. *Table* *S2* provided the sequences of the primers used for qRT‐PCR.

### Western blotting

Western blotting procedure was performed according to literature report.^18^ Briefly, skeletal muscle samples or treated C2C12 cells were lysed in ice‐cold RIPA buffer with the PMSF protease inhibitor (ST505, Beyotime, China). The total protein concentration of the supernatant was determined using a BCA assay kit (P0012S, Beyotime, China). Equal amounts of proteins from different groups were resolved via 8% or 10% SDS–PAGE and then transferred onto PVDF membranes (IPVH00010, Merck Millipore, USA). Subsequently, the membranes were blocked for 2 h at 37°C with 5% milk in TBST and then incubated overnight at 4°C with primary antibodies against PDHB (14744‐1‐AP, Proteintech, 1:2000), MyoD (18943‐1‐AP, Proteintech, 1:1000), MyoG (ab1835, Abcam, 1:1000), MyHC (MF‐20, DSHB, 1:1000) and β‐actin (AC026, ABclonal, 1:1000). Then, the membranes were washed three times with TBST and incubated with horseradish peroxidase‐conjugated anti‐rabbit or anti‐mouse IgG antibodies (AS003 or AS014, ABclonal, 1:1000) at temperature for 1 h. The target protein bands were visualized using an enhanced chemiluminescence high‐sensitivity reagent (JN.BIOTOOLS, Wuxi, China) and a Bio‐Rad ChemiDoc Imager (Bio‐Rad). The densitometric values were determined using the ImageJ software (National Institutes of Health, USA). The relative protein levels were normalized against the levels of β‐actin, used as an internal control.

### Liquid chromatography–mass spectrometry untargeted metabolomics

C2C12 cells were transfected with si‐NC or si‐PDHB and then induced to differentiate in DM medium for 3 days. For untargeted metabolomics detection, differentiated C2C12 cells were washed three times with PBS, and then 1 mL methanol/acetonitrile/water (2:2:1 volume) solution was added to each well. Afterward, they were scraped off quickly and quenched in liquid nitrogen. Next, the samples were vortexed and then subjected to ultrasonication at 4°C to pellet the cell debris and proteins and subsequently centrifuged at 14 000 rpm for 15 min at 4°C. The supernatants were collected and then dried in a vacuum centrifuge. For liquid chromatography–mass spectrometry (LC–MS) untargeted metabolomics analysis, the samples were re‐dissolved in 150 μL methanol/water (4:1, v/v) solvent. Metabolic profiles were measured through using Vanquish UHPLC system (Thermo Fisher) equipped with an Orbitrap Q Exactive series mass spectrometer (Thermo Fisher). Waters UPLCBEH C18 column (1.7 μm, 2.1 × 100 mm) was applied. Mobile phases A and B were carried out using water and acetonitrile/methanol (2/3, v/v), respectively, both containing 0.1% formic acid. Raw LC–MS data files were processed using Compound Discoverer 3.1 (CD3.1, Thermo Fisher) to conduct peak alignment, peak picking and quantitation of the metabolites. Metabolomics data and enrichment analyses, and multivariate data analysis, such as orthogonal partial least squares discriminant analysis (OPLS‐DA), were performed using MetaboAnalyst Version 5.0 (http://www.metaboanalyst.ca). In the OPLS‐DA model, variable importance in the projection (VIP) value of each variable was calculated to determine the corresponding contribution to the classification. The metabolites with the standard of VIP value >1 were subjected to Student's *t* test for identifying the significance of each metabolite, and *P* values <0.05 were regarded to signify statistical significance.

### RNA sequencing and data analysis

RNA sequencing (RNA‐Seq) and data analysis were performed as previously reported.^19^, ^20^, ^21^ Briefly, an RNA extraction kit (K101, JN.BIOTOOLS, Wuxi, China) was used to extract RNA from the C2C12 cells transfected with si‐NC or si‐PDHB. For RNA‐Seq, the RNA quality and integrity were examined using Bioanalyzer 2100 (Agilent). After preparing the cDNA library, RNA‐Seq was conducted by Genewiz (Suzhou, China) by using Illumina Novaseq (paired‐end, 150 bp, PE150). Raw data files (.fastq) were mapped to the mouse reference genome Mus_musculus GRCm38 using the STAR package (Version 2.7). Finally, to identify the differentially expressed genes (DEGs), FPKM was calculated using StringTie, EdgeR and DESeq2. DEGs were defined as transcripts with a standard of log_2_ (fold change) >1 or <−1 and statistical significance (*P* value <0.05). The Metascape database (http://metascape.org/) was used for the Gene Ontology (GO) analysis.

### Dual‐luciferase reporter assay

For predicting the binding sites of FoxP1 and Arih2, the JASPAR database (http://www.jaspar.genereg.net) was applied. The promoter region p(−2000/+180) of *Arih2* was amplified via PCR and cloned into the pGL‐4.10 basic vector (Transheep, Shanghai, China). C2C12 cells were co‐transfected with Arih2‐p(−2000/+180) Luc and the pcDNA3.1–FoxP1 overexpression construct by using jetPRIME transfection reagent (Polyplus). The dual‐luciferase reporter assay was carried out using Dual‐Luciferase Reporter Assay System (DD1205‐01, Vazyme, Nanjing, China). Renilla luciferase activity was used to evaluate transfection efficiency.

### Statistical analysis

All the data were expressed as mean ± SD and subjected to one‐way ANOVA or Student's *t* test by using the GraphPad Prism 8.0 software. *P* values <0.05 were considered to indicate statistical significance. This study collected data from at least three biological replicates.

## Results

### Pyruvate dehydrogenase B is down‐regulated in the muscle during ageing whereas up‐regulated during myogenic differentiation

**Figure 1:**
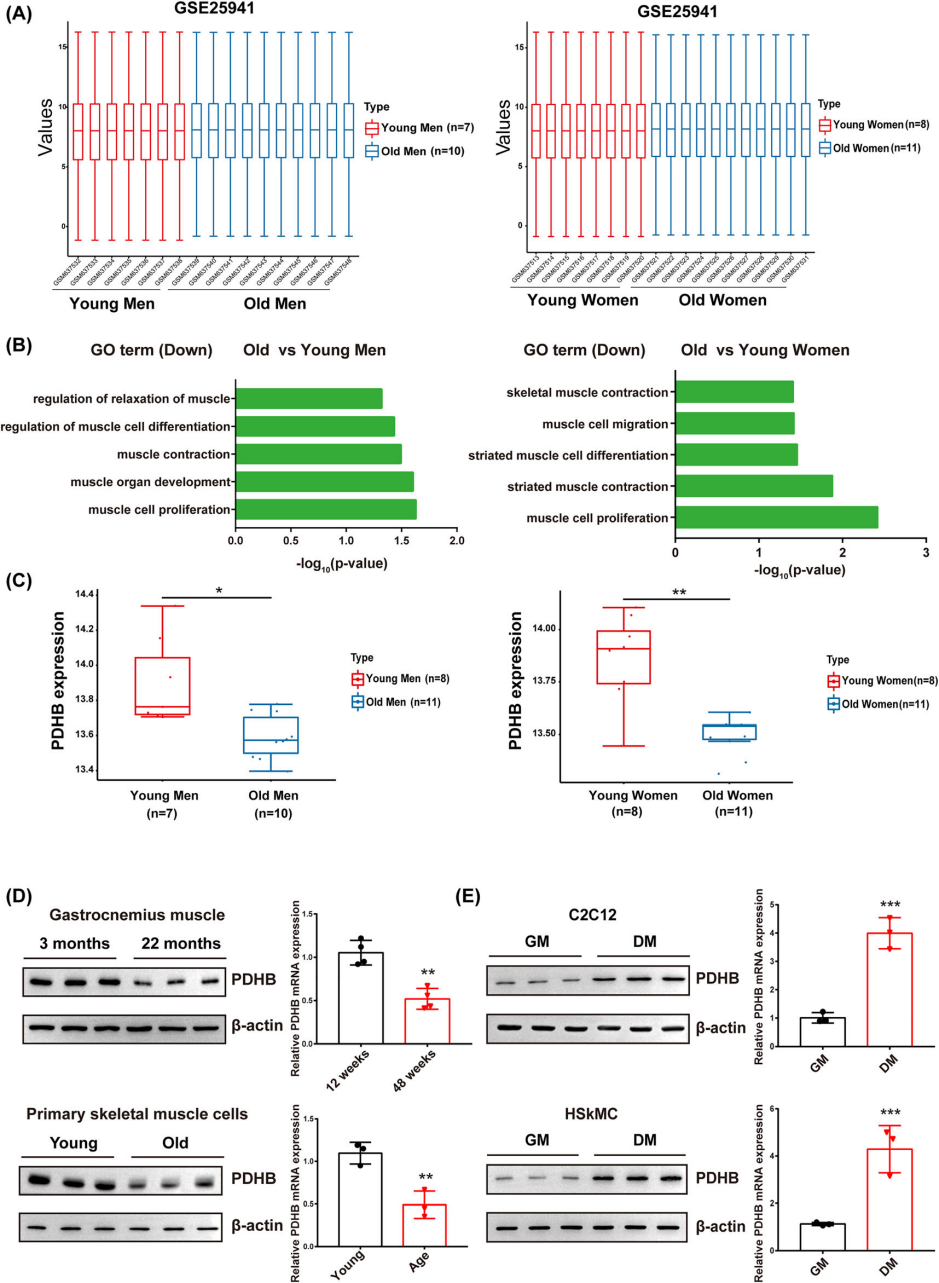
PDHB is up‐regulated during myogenic differentiation and down‐regulated during ageing. (A) Box plot of the gene expression data from men (left) or women (right) in GSE25941 following standardization. (B) From data on young and old men (left) or women (right), down‐regulated GO term enrichment analysis related to skeletal muscle function was performed. (C) PDHB expression levels in young and old men (left) or women (right) in GSE25941. (D) Western blotting and qRT‐PCR analyses of PDHB protein and mRNA levels in skeletal muscle from 3‐ and 22‐month‐old mice (*n* = 4, per group) and in primary skeletal muscle cells from young and ageing mice. (E) PDHB protein and mRNA levels in C2C12 cells and HSkMC grown in growth medium (GM) and differentiation medium (DM). β‐Actin was used as a loading control. Data are shown as mean ± SD. ^*^ *P* < 0.05, ^**^ *P* < 0.01 and ^***^ *P* < 0.001 (Student's *t* test)

To evaluate the relationship between ageing and skeletal muscle physiology in humans and to find novel targets, we analysed the microarray data from the GEO database. The microarray gene expression dataset GSE25941 includes samples from 15 young people (25 ± 1 years old, 7 men and 8 women) and 21 old people (78 ± 1 years old, 10 men and 11 women). The skeletal muscle biopsies were obtained from the vastus lateralis at the basal state. As shown in *Figure* *1A*, the data were neatly distributed after background adjustment and normalization. We next performed GO analysis on the DEGs. The GO enrichment analysis showed that GO terms related to skeletal muscle physiological functions, such as muscle relaxation and muscle contraction, were down‐regulated during ageing in men and women (*Figure* *1B*).

Additionally, the expression of PDHB was significantly reduced in the skeletal muscle samples from the elderly versus those from the young people (*Figure* *1C*). Furthermore, to assess the expression pattern of PDHB in the skeletal muscle of mice during ageing, we analysed PDHB mRNA and protein levels via qRT‐PCR and western blotting, respectively, in young (3 months old) and old (22 months old) C57BL/6J mice. Consequently, we found that PDHB expression markedly decreased during skeletal muscle ageing in the mouse (*Figure* *1D*). Decreased PDHB expression in the ageing state was also confirmed by extracting and culturing primary myoblasts from young and old mice. These findings reveal that PDHB might contribute to the physiological process of skeletal muscle ageing.

Previous studies have shown that the decline in myogenic differentiation is the most important physiological change in skeletal muscle ageing. To explore the potential role of PDHB in skeletal muscle differentiation, we first used the C2C12 and primary human skeletal muscle cell line (HSkMC) myoblast differentiation models, incubating C2C12 and HSkMC myoblasts and allowing them to differentiate into myotubes. As shown in *Figure* *1E*, the protein and mRNA levels of PDHB were significantly increased during this process. Additionally, we found that PDHB mRNA and protein levels were up‐regulated in CTX‐induced skeletal muscle regeneration in mice (*Figure* *S1* *A*–*C*). Altogether, these results indicate that PDHB may be associated with ageing‐related muscular physiology and involved in the process of myogenic differentiation.

### Pyruvate dehydrogenase B deficiency impairs myogenic differentiation

**Figure 2:**
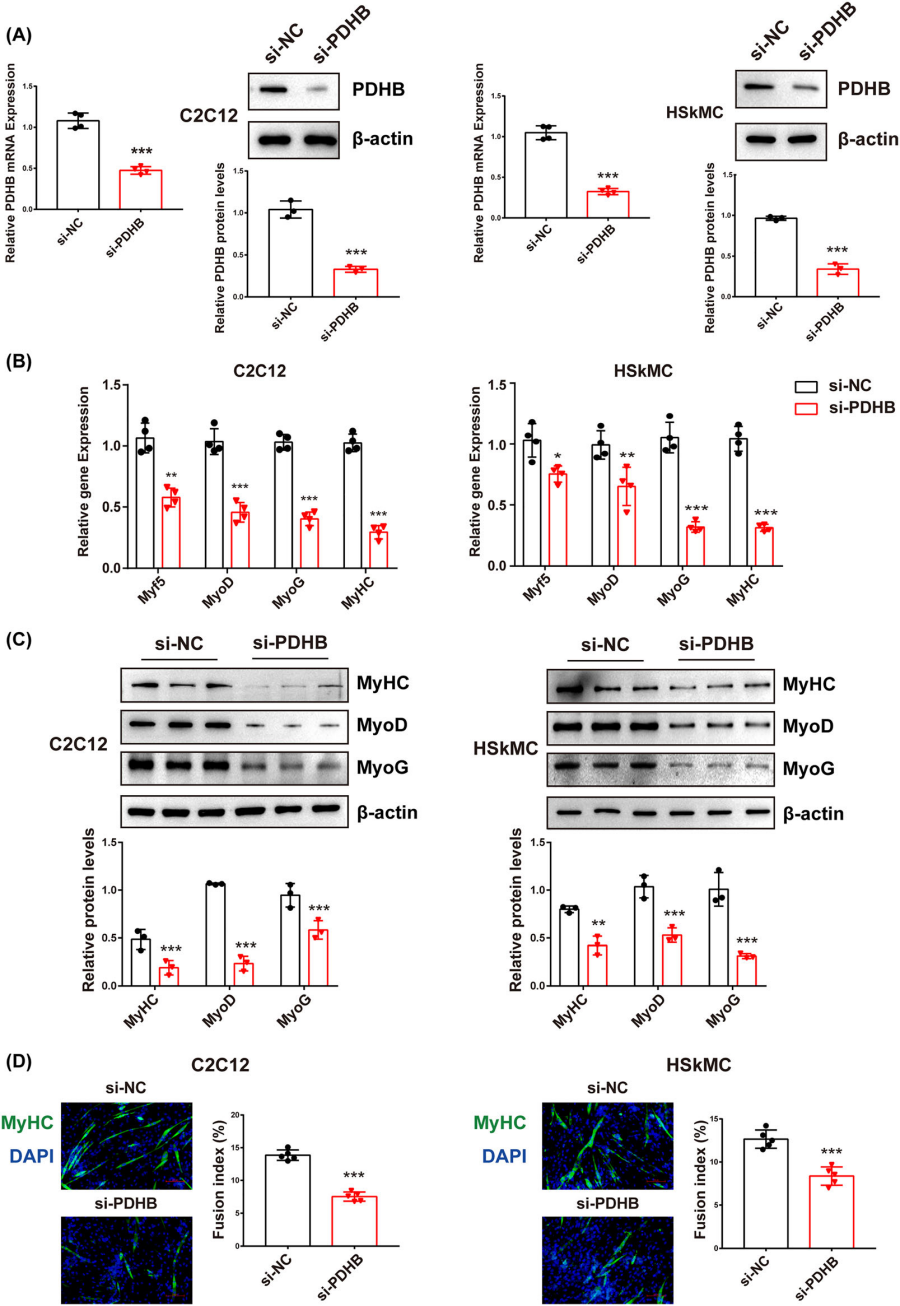
PDHB knockdown impairs myogenic differentiation. C2C12 cells and HSkMC were transfected with si‐NC or si‐PDHB and then induced to differentiate in the differentiation medium (DM) for 3 or 5 days. (A) The mRNA and protein levels of PDHB in C2C12 cells and HSkMC. (B) qRT‐PCR was performed to detect the mRNA expressions of myogenic differentiation markers (*Myf5*, *MyoD*, *MyoG* and *MyHC*) in C2C12 cells and HSkMC. (C) Western blotting showing the protein levels of MyHC, MyoD, MyoG and β‐actin in C2C12 cells and HSkMC. The relative protein levels of the target proteins were normalized to those of β‐actin. (D) MyHC (green) immunofluorescence staining was used to assess the myotube formation of C2C12 cells and HSkMC transfected with si‐NC or si‐PDHB. The cell nucleus was stained with DAPI (blue). Scale bar = 100 μm. The fusion index (the percentage of nuclei in fused myotubes out of the total nuclei) was calculated. Data are shown as mean ± SD. ^*^ *P* < 0.05, ^**^ *P* < 0.01 and ^***^ *P* < 0.001 (Student's *t* test) versus the si‐NC group

To further elucidate the function of PDHB in myogenic differentiation, we knocked down PDHB in C2C12 myoblasts and HSkMC via siRNA transfection (si‐PDHB) and then stimulated myotube formation. Both qRT‐PCR and western blot data demonstrated that PDHB siRNA transfection remarkably reduced the expression of PDHB (*Figure* *2A*). Moreover, PDHB knockdown substantially reduced the mRNA levels of myogenic differentiation marker genes, including *Myf5*, *MyoD*, *MyoG* and *MyHC*, after differentiation induction (*Figure* *2B*). In addition, the protein levels of MyoD, MyoG and MyHC also declined considerably (*Figure* *2C*). Consistently, immunofluorescence analysis for MyHC showed that myogenic differentiation was impaired and cell fusion index markedly declined upon PDHB knockdown in both C2C12 myoblasts and HSkMC (*Figure* *2D*). Overall, these findings indicated that PDHB deficiency impaired myogenic differentiation.

### Knockdown of pyruvate dehydrogenase B reduces pyruvate metabolism and leads to lactate accumulation

**Figure 3:**
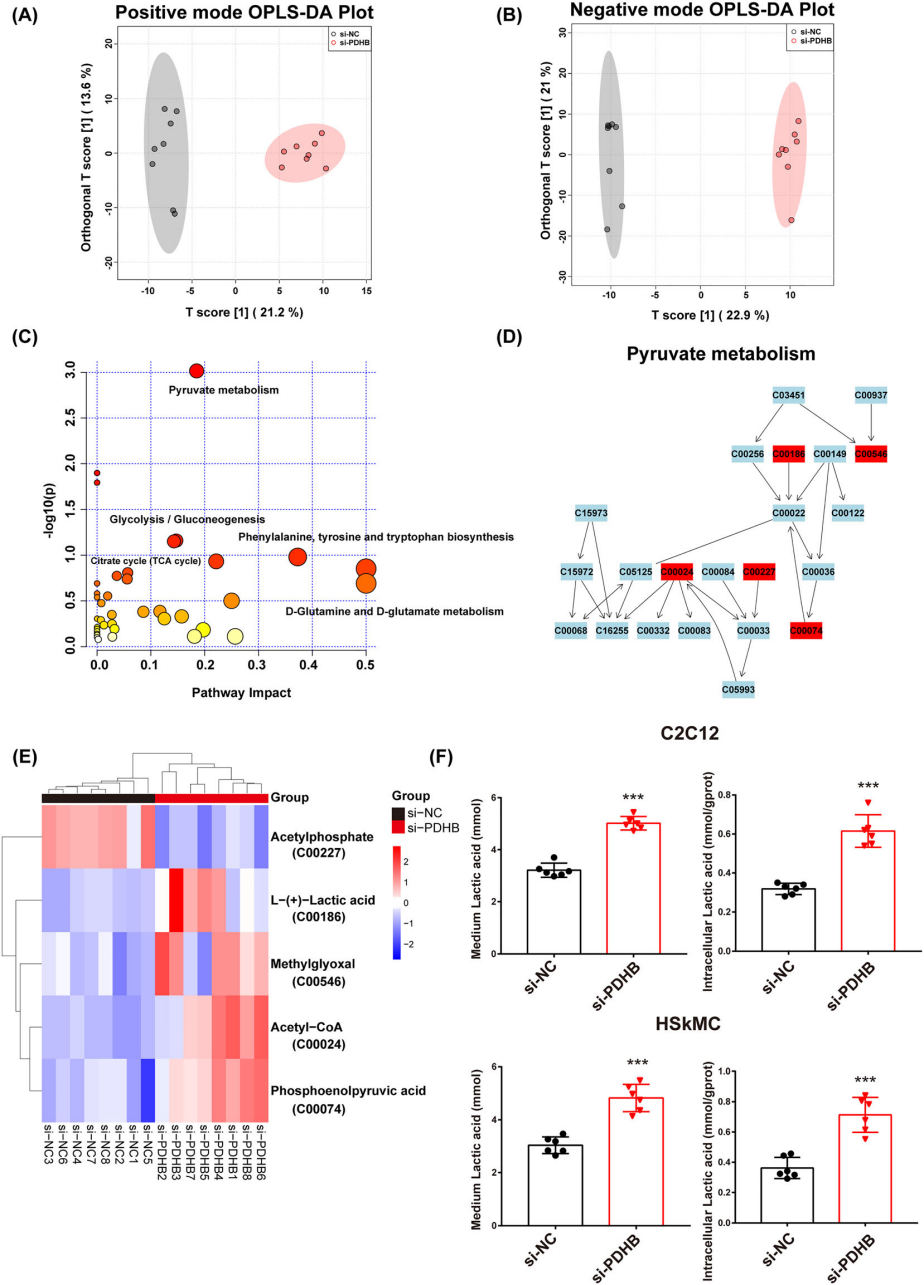
Metabolomic analysis. (A) Positive‐mode OPLS‐DA score plot. (B) Negative‐mode OPLS‐DA score plot. (C) Integrative metabolic pathway enrichment analysis performed using MetaboAnalyst 5.0. (D) Schematic overview of the differential metabolites involved in pyruvate metabolism. Red represents the significantly changed metabolites (VIP > 1, *P* < 0.05). (E) Heatmap showing the differential metabolites from (D). (F) Lactic acid levels in the supernatant and cells of C2C12 (up) and HSkMC (down) cultures. Data were expressed as mean ± SD and analysed using Student's *t* test. ^*^ *P* < 0.05, ^**^ *P* < 0.01 and ^***^ *P* < 0.001 versus the si‐NC group; *n* = 6–8

PDHB located in mitochondria is involved in multiple energy metabolism pathways. To explore the potential effects of PDHB on the metabolic processes in myogenic differentiation, we conducted LC–MS‐based untargeted metabolomics to analyse the cellular metabolites after PDHB knockdown. In total, 816 metabolites (335 in the positive mode and 481 in the negative mode) were identified. In addition, to confirm our metabolomics results, we comprehensively analysed the metabolomics data by using the MetaboAnalyst 5.0 software. The OPLS‐DA results for the detected metabolites are presented in *Figure* *3A* (positive mode) and *Figure* *3B* (negative mode). The two groups were distinctly separated into two differentiated clusters, suggesting that PDHB knockdown caused a significant change in the overall metabolite profile. Furthermore, significantly enriched functional categories, such as pyruvate metabolism, glycolysis/gluconeogenesis, citrate cycle (TCA cycle) and pyruvate metabolism, were observed among the metabolic pathways disturbed upon PDHB knockdown (*Figure* *3C*). In addition, the comprehensive metabolomic pathway of pyruvate metabolism is shown to identify remarked relationships among these metabolites (*Figure* *3D*). Additionally, l‐(+)‐lactic acid, methylglyoxal, acetyl‐CoA and phosphoenolpyruvic acid were up‐regulated, whereas acetylphosphate was down‐regulated, upon PDHB knockdown (*Figure* *3E*). Among these, l‐(+)‐lactic acid was the most significantly affected metabolite.

Next, we measured intracellular and medium lactate levels of C2C12 and HSkMC using a lactate assay kit. As shown in *Figure* *3F*, both lactate levels were significantly elevated upon PDHB knockdown. Collectively, these results demonstrated that PDHB knockdown mainly affected pyruvate metabolism and resulted in lactic acid accumulation.

### Transcriptomic analysis indicates that pyruvate dehydrogenase B modulates cellular catabolic processes

**Figure 4:**
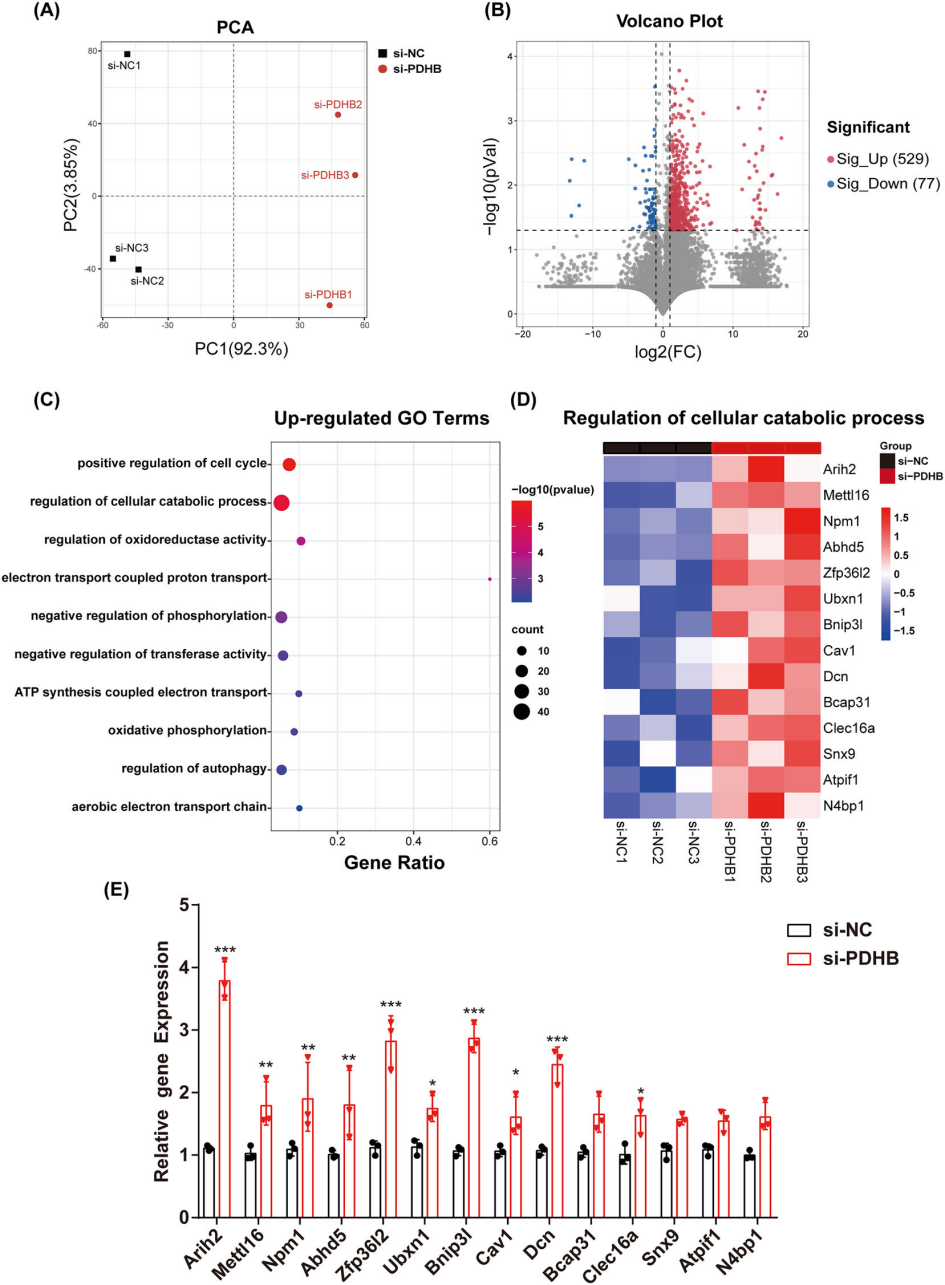
Transcriptomic analysis. (A) Principal component analysis (PCA) of the RNA‐sequencing (RNA‐Seq) data from C2C12 cells. Two groups of samples (si‐NC, black; si‐PDHB, red) are shown on the PCA plot. (B) The volcano plot of the RNA‐Seq data. The red and blue data points represent the up‐regulated (log_2_FC > 1, *P* < 0.05) and down‐regulated (log_2_FC < −1, *P* < 0.05) genes, respectively. (C) Gene ontology analysis of the up‐regulated DEGs. (D) Heatmap showing representative genes involved in the regulation of cellular catabolic process (the fold change decreases from the top towards the bottom). (E) qRT‐PCR was performed to calculate the mRNA levels of the genes shown in (D). Data were expressed as mean ± SD and analysed using Student's *t* test. ^*^ *P* < 0.05, ^**^ *P* < 0.01 and ^***^ *P* < 0.001 versus the si‐NC group; *n* = 3

To investigate the action mechanism of PDHB in myogenic differentiation, the gene expression profiles of C2C12 cells with or without PDHB knockdown were identified via RNA‐Seq. In the principal component analysis (PCA), the control (si‐NC) cells were clearly distinguishable from the PDHB‐knocked down (si‐PDHB‐transfected) cells (*Figure* *4A*). A total of 606 DEGs (log_2_|fold change| > 1 and *P* value <0.05) were identified, of which 77 were down‐regulated and 529 were up‐regulated (*Figure* *4B*). GO enrichment analysis showed that the significantly enriched functional categories among the up‐regulated genes included positive regulation of cell cycle, regulation of cellular catabolic processes and regulation of oxidoreductase activity (*Figure* *4C*). Among these categories, the regulation of cellular catabolic process pathway contained the largest number of up‐regulated genes. Nevertheless, no particularly enriched biological process, except for muscle cell differentiation and carboxylic acid metabolic process, was found among the down‐regulated genes. These data suggested that the regulation of cellular catabolic processes is the main pathway affected by the PDHB knockdown.

To identify the genes linking PDHB to the regulation of cellular catabolic processes, we displayed the main representative up‐regulated genes on a heatmap and the fold change decreased gradually from top to bottom (*Figure* *4D*). Additionally, the changes in gene expression levels upon PDHB knockdown were confirmed via qRT‐PCR (*Figure* *4E*). *Arih2*, *Zfp36l2*, *Bnip3l* and *Dcn* showed the most significant up‐regulation (*P* value <0.001) in the si‐PDHB group versus the si‐NC group.

In summary, these results showed that PDHB knockdown might inhibit myogenic differentiation by up‐regulating the cellular catabolic pathway, and the main genes whose expression was affected by PDHB knockdown are *Arih2*, *Zfp36l2*, *Bnip3l* and *Dcn*.

### *Arih2* silencing rescues pyruvate dehydrogenase B knockdown‐mediated inhibition of myogenic differentiation

**Figure 5:**
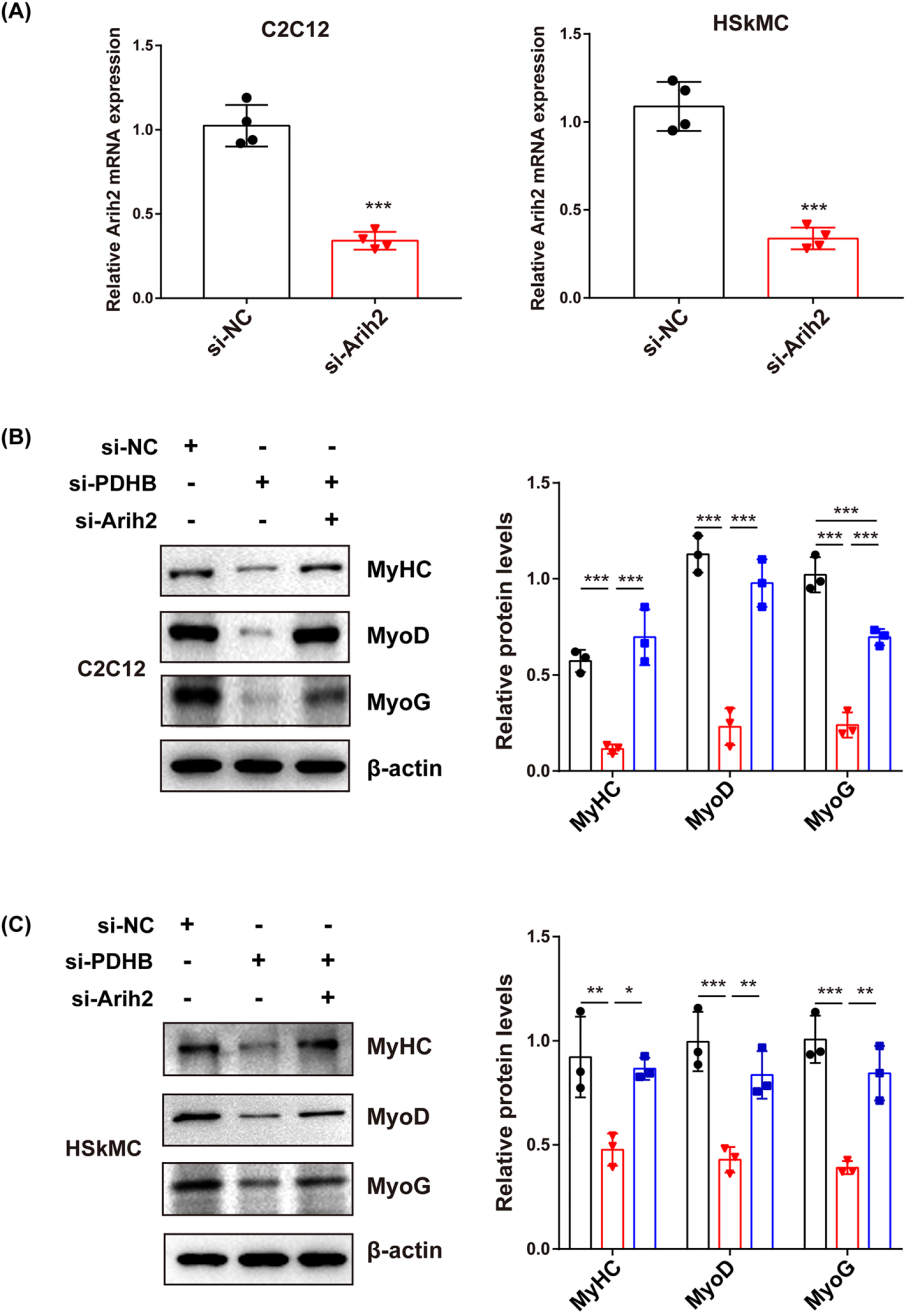
Arih2 is involved in PDHB‐mediated myogenesis. (A) The mRNA expression of Arih2 in C2C12 cells (left) and HSkMC (right) transfected with si‐Arih2. Western blot and quantification of MyHC, MyoD and MyoG in the three groups from (B) C2C12 cells and (C) HSkMC. β‐Actin was used as the loading control. The results of the grayscale analysis are on the right. Data were expressed as mean ± SD and analysed using Student's *t* test or one‐way ANOVA. ^*^ *P* < 0.05, ^**^ *P* < 0.01 and ^***^ *P* < 0.001

To investigate which one of the four genes (*Arih2*, *Zfp36l2*, *Bnip3l* and *Dcn*) is the most pivotal in the myogenic function of PDHB, each gene was knocked down in C2C12 cells. The changes in lactate accumulation and expression of myogenic differentiation proteins due to PDHB knockdown were rescued by Arih2 knockdown (*Figures* *5A*–*C* and *S4* *D*). However, knocking down other catabolism‐related proteins (Dcn, Bnip3l and Zfp36l2) did not have the same remarkable effect (*Figure* *S2* *A*–*F*). Together, these results demonstrated that PDHB and Arih2 function in the same pathway to restrict myogenic differentiation and Arih2 is a key downstream regulator of PDHB in myogenic differentiation.

### Pyruvate dehydrogenase B regulates Arih2 transcription via FoxP1

**Figure 6:**
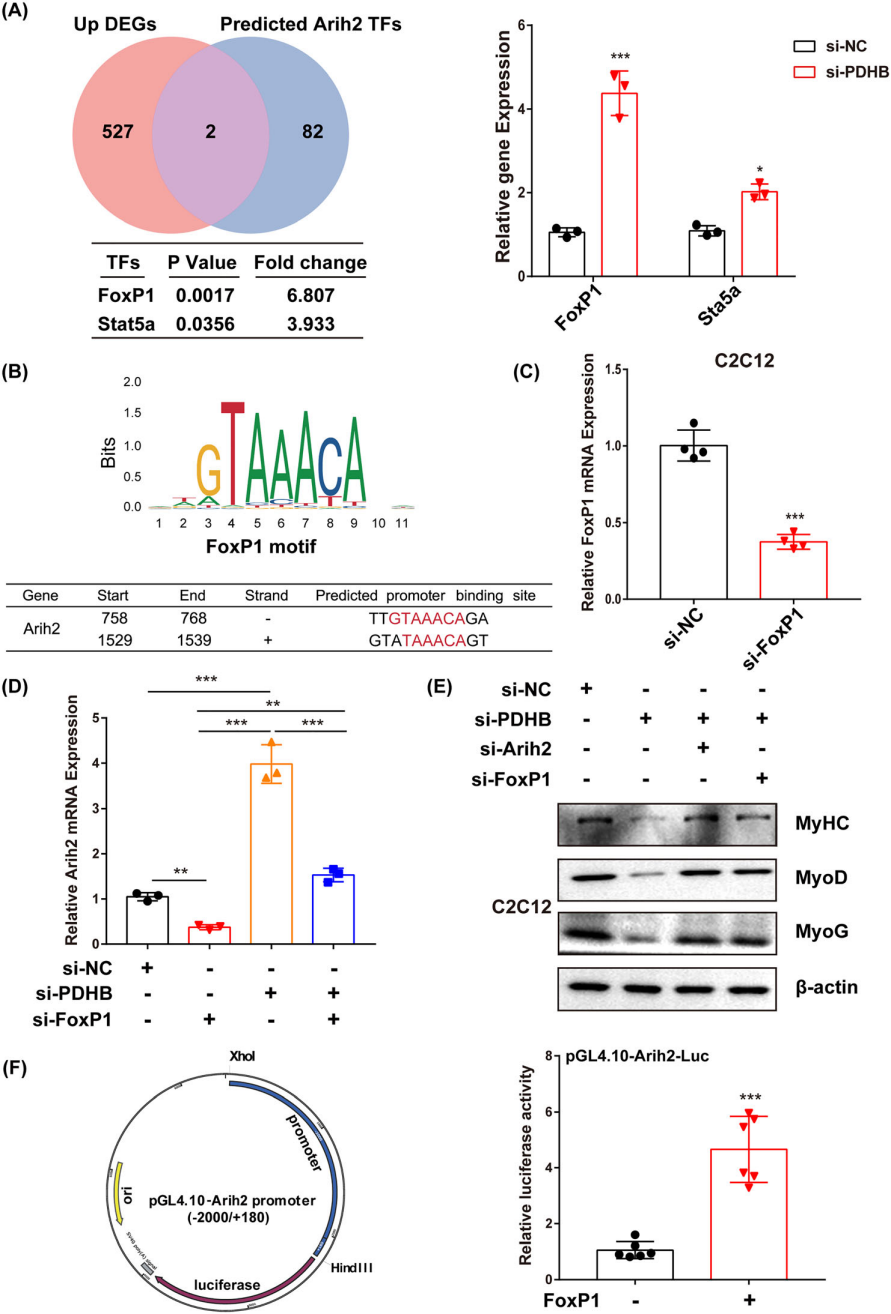
Prediction of the transcription factors (TFs) regulating *Arih2* expression and experimental validation of the Arih2 function in myogenesis. (A) Venn plot showing the intersection between the up‐regulated DEGs and predicted TFs regulating *Arih2* expression. *FoxP1* and *Stat5a* mRNA levels were measured via qRT‐PCR. (B) TF motif enrichment analysis results from the JASPAR database. qRT‐PCR was performed to measure the mRNA levels of (C) *FoxP1* and (D) *Arih2*. (E) Western blotting showing the protein levels of MyHC, MyoD, MyoG and β‐actin. (F) Dual‐luciferase reporter assay result. C2C12 cells were transfected with a FoxP1‐expression plasmid or empty vector, plus an Arih2‐dependent luciferase reporter to assess Arih2 activity. Data were expressed as mean ± SD and analysed using Student's *t* test or one‐way ANOVA. ^*^ *P* < 0.05, ^**^ *P* < 0.01 and ^***^ *P* < 0.001

To uncover potential TFs that control Arih2 transcription, a Venn diagram was prepared to examine the intersection between the up‐regulated DEGs and the TFs regulating *Arih2* that are predicted via the JASPAR database. As shown in *Figure* *6A*, FoxP1 and Stat5a were identified, and qRT‐PCR analysis confirmed that *FoxP1* and *Stat5a* mRNA levels were remarkably increased in C2C12 cells upon PDHB knockdown. These results suggested that PDHB modulates Arih2 through FoxP1 or Stat5a in myogenic differentiation. Further analyses showed that FoxP1 knockdown alone or alongside PDHB knockdown strongly inhibited Arih2 transcription in C2C12 cells (*Figure* *6C*,*D*). Accordingly, FoxP1 may be a TF regulating *Arih2*, and thus, the possible binding sites of FoxP1 on the *Arih2* promoter were predicted using the JASPAR database (*Figure* *6B*). However, Stat5a knockdown had little effect according to the qRT‐PCR and western blot analyses (*Figure* *S3* *A*–*C*), and these results were confirmed at the protein level (*Figure* *6E*).

To validate the role of FoxP1 in *Arih2* transcription, a luciferase reporter containing the Arih2 promoter sequence was constructed. Co‐transfection of the reporter and *Foxp1* activated luciferase transcription and activity (*Figure* *6F*). These results were also validated using HSkMC (*Figure* *S4* *A*–*C*). Altogether, these results demonstrated that PDHB regulates *Arih2* transcription via FoxP1 during skeletal muscle differentiation.

### Pyruvate dehydrogenase B overexpression promotes myogenic differentiation and improves muscle function of ageing mice

**Figure 7:**
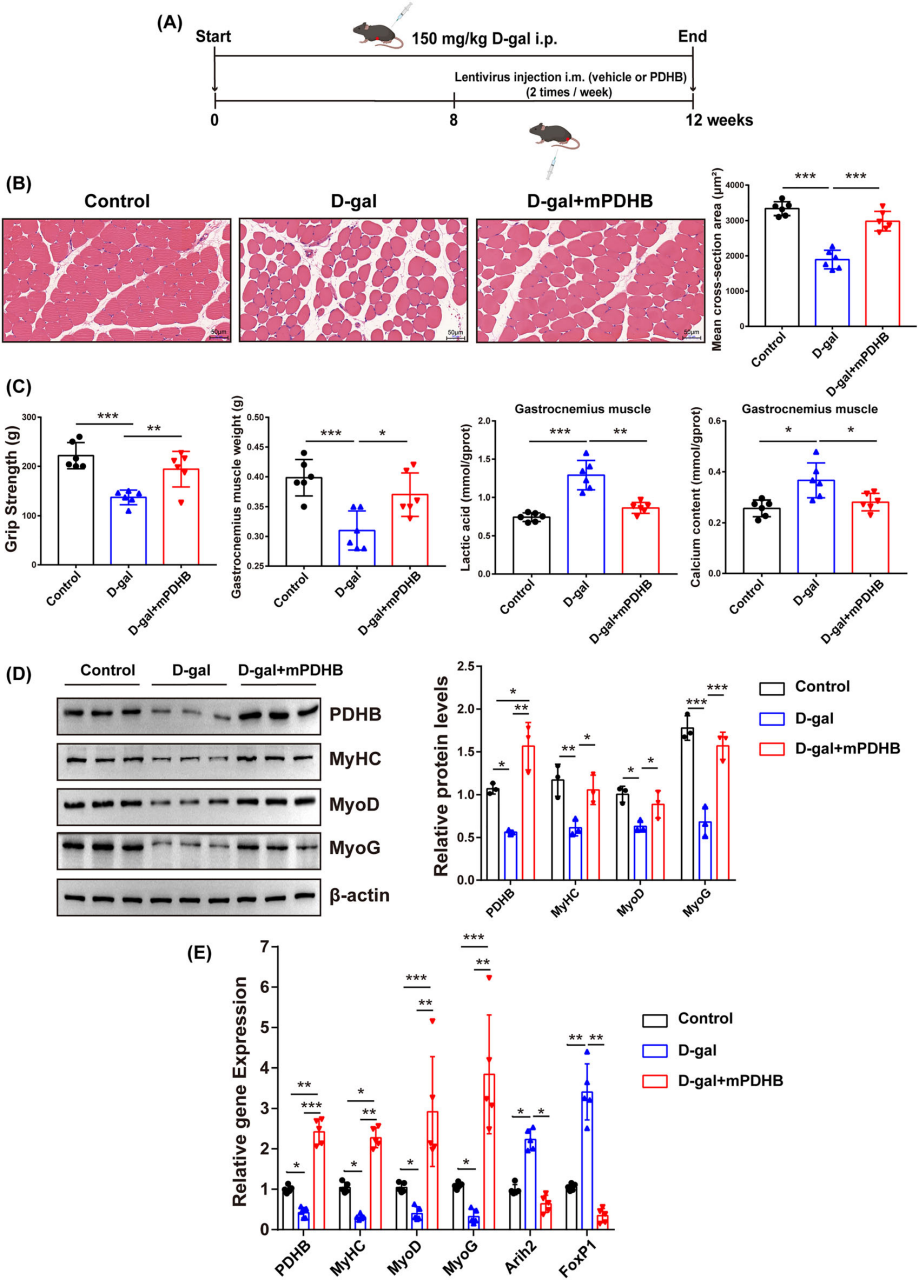
Overexpression of PDHB promotes myogenic differentiation and improves muscle mass and function in ageing mice. (A) Schematic diagram of the mice experiment. (B) H&E staining of gastrocnemius muscle fibre (magnification 200×, scale bar = 50 μm), and the cross‐sectional area (CSA) of muscle fibres measured by Image‐Pro Plus software. (C) Grip strength, gastrocnemius muscle weight, contents of lactic acid and calcium in gastrocnemius muscles of mice from the three groups. (D) Western blotting showing the protein levels of MyHC, MyoD and MyoG. β‐Actin was used as a loading control to quantify the relative protein levels. (E) mRNA level of *PDHB*, *MyHC*, *MyoD*, *MyoG*, *Arih2* and *FoxP1* in gastrocnemius muscle from the three groups. Data were expressed as mean ± SD and analysed using Student's *t* test or one‐way ANOVA, ^*^ *P* < 0.05, ^**^ *P* < 0.01 and ^***^ *P* < 0.001; *n* = 6 mice per group

We further conducted an in vivo study to explore whether overexpression of PDHB promotes myogenic differentiation and improves sarcopenia. As shown in *Figure* *7A*, d‐gal‐induced aged mice were injected intramuscularly with the lentivirus encoding PDHB. It was found that PDHB overexpression significantly increased mean muscle fibre CSA (*Figure* *7B*). In addition, several features of d‐gal‐induced sarcopenia, such as loss of grip strength, decreased gastrocnemius muscle mass and accumulation of lactic acid and calcium, were partially ameliorated by PDHB overexpression (*Figure* *7C*). Injection of PDHB‐overexpressing lentivirus resulted in a significant increase in gastrocnemius PDHB expression compared with controls, whereas d‐gal‐induced ageing mice showed significantly decreased PDHB expression in gastrocnemius (*Figure* *7D*,*E*). Moreover, the myogenic markers (MyoD, MyoG and MyHC) were decreased in the d‐gal group but were restored by PDHB overexpression (*Figure* *7D*). Compared with the control group, the expressions of Arih2 and FoxP1 were significantly up‐regulated in the d‐gal group, whereas overexpression of PDHB inhibited the activation of the FoxP1–Arih2 axis (*Figure* *7E*).

The in vitro study further confirmed the above results. C2C12 cells transfected with the pcDNA3.1 vector or pcDNA3.1–PDHB plasmid were used to evaluate the contribution of PDHB to the differentiation of myoblasts in vitro. Both qRT‐PCR and western blot data confirmed that PDHB was significantly overexpressed after pcDNA3.1–PDHB transfection (*Figure* *S5* *A*). The mRNA and protein levels of myogenic TFs (Myf5, MyoD and MyoG) and the protein level of MyHC, serving as a terminal myogenic differentiation marker, were significantly elevated in PDHB‐overexpressing C2C12 cells (*Figure* *S5* *B*,*C*). Similarly, data from immunofluorescence analyses demonstrated that the PDHB‐overexpressing C2C12 myoblasts formed larger myotubes and displayed a higher fusion index than the control cells (*Figure* *S5* *D*). Collectively, these results confirmed that PDHB overexpression promoted myogenic differentiation and improved muscle function of ageing mice in vivo and in vitro.

## Discussion

**Figure 8:**
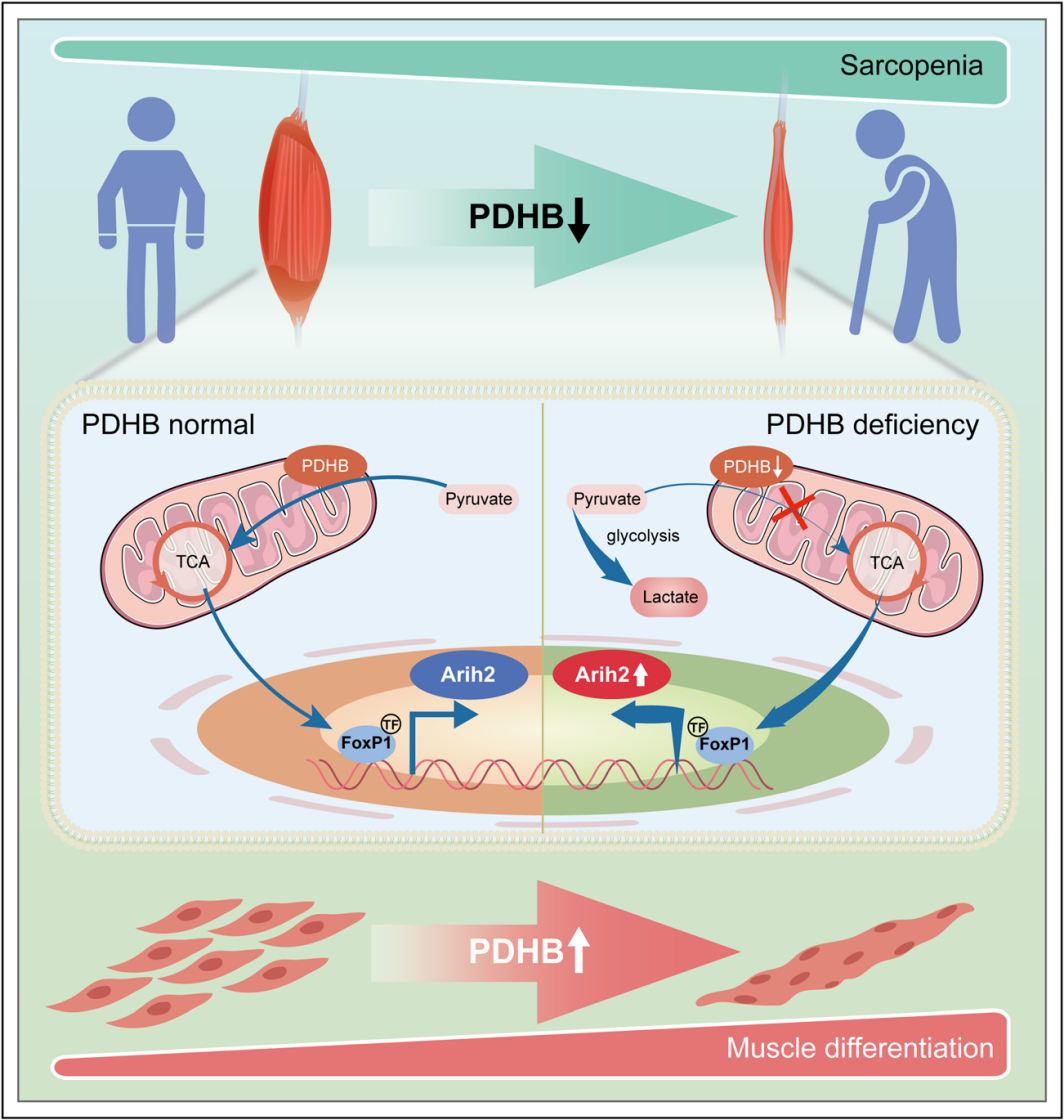
Schematic presentation of the PDHB–FoxP1–Arih2 axis

The ageing‐related progressive loss of skeletal muscle mass, known as sarcopenia, is the most common type of muscle atrophy, which induces a profound impact on the life quality and mortality of elderly people. However, molecular pathological mechanisms involved in muscle ageing remain largely unknown. Improvement of skeletal muscle differentiation is an effective way to alleviate sarcopenia.^22^ The results of this work, as illustrated in *Figure* *8*, highlight the crucial role of PDHB‐mediated FoxP1–Arih2 axis in myogenesis. Thus, the PDHB signalling contributes to skeletal muscle differentiation and can be targeted as a potential therapy in sarcopenia.

Ageing leads to a decline in the capacity of skeletal muscle differentiation and significant down‐regulation of related genes. In this work, we found that PDHB expression was reduced in elderly skeletal muscle and primary skeletal muscle cells. Thus, the hypothesis that PDHB is involved in skeletal muscle differentiation was proposed. Increasing evidence has shown that skeletal muscle differentiation is a complicated process predominantly regulated by MRFs. MyoD and MYF5 participate in the first stage of skeletal muscle development by promoting the proliferation of myogenic progenitor cells and the differentiation of these cells into myoblasts.^23^ Myogenin plays a critical role in the differentiation of myoblasts into myotubes,^24^ and MyHC is considered a terminal marker of myogenic differentiation.^25^ In this work, we confirmed that PDHB was up‐regulated during skeletal muscle differentiation in vivo and in vitro through a CTX‐induced muscle regeneration model and a cell culture model based on differentiation of C2C12 and HSkMC myoblasts, respectively. Furthermore, PDHB knockdown or overexpression showed a decreased or increased expression of Myf5, MyoD, MyoG and MyHC, which would lead to a lower or higher myogenic differentiation rate. Moreover, PDHB overexpression in skeletal muscle alleviated d‐gal‐induced sarcopenia in mice. Altogether, these data suggest that PDHB promotes the myogenic differentiation process.

Studies have shown that PDHB encodes a pyruvate dehydrogenase that catalyzes the conversion of pyruvate to acetyl‐CoA for the TCA cycle.^26^ PDHB plays metabolic regulatory roles in various organs and cells. Here, we characterized the overall effect of PDHB on the metabolic profile during myogenic differentiation via metabolomics analysis. The knockdown of PDHB resulted in abnormal pyruvate metabolism. Specifically, pyruvate was not metabolized through the TCA cycle, but shifted towards the glycolytic pathway, eventually leading to the production of large amounts of lactate. It is also widely presumed that lactic acid accumulation reduces myogenic differentiation capacity.^27^ Our previous study also confirmed the detrimental effect of lactate accumulation on myogenic differentiation and the down‐regulation of PDHB expression during this process.^28^ How PDHB regulates glycolysis and lactate production in the skeletal muscle needs further study.

Transcriptomic analysis showed that most of the DEGs upon PDHB knockdown were up‐regulated. Additionally, GO term enrichment analysis indicated that positive regulation of cell cycle, regulation of cellular catabolic processes and regulation of oxidoreductase activity were the mainly enriched pathways among the DEGs. In the process of skeletal muscle myogenesis, myoblasts undergo rapid proliferation, followed by the cell‐cycle exit, myogenic differentiation and, ultimately, cell fusion. Cell‐cycle exit is necessary for cellular differentiation.^29^ Interestingly, PDHB knockdown promoted the cell cycle in C2C12 cells and thus inhibited the differentiation process. Moreover, numerous studies have reported that cellular catabolic processes such as autophagy are essential for cell survival, differentiation, development and maintaining homeostasis.^30^

Ageing causes a remarkable reduction in muscle mass due to a shift towards protein degradation from protein synthesis.^31^ The ubiquitin–proteasome system (UPS) is a major pathway of protein degradation in mammalian cells. UPS selectively degrades target proteins following their ubiquitination by various E3 ligases. Many studies have indicated that the enzymatic activities of ubiquitin E3 ligases play a particularly critical role in regulating skeletal muscle mass.^32^, ^33^ A study has revealed that congenital deficiencies of the human pyruvate dehydrogenase complex involve a post‐translational modification and result in enhanced proteasome‐mediated degradation caused by increased ubiquitination.^26^ In the present study, we identified Arih2 as a downstream gene of PDHB and verified that Arih2 knockdown rescued the myogenic differentiation inhibited by PDHB knockdown. Arih2 belongs to a RING1‐in‐between‐RING2 (RBR) E3 ligase family, and studies have recently reported that Arih2 is involved in ageing‐associated muscle degeneration and ubiquitin‐dependent modification in the skeletal muscle.^34^, ^35^, ^36^ Additionally, a study has indicated that E3 ligases are involved in muscle atrophy through the regulation of the TF forkhead box O (FoxO).^37^ Here, we tested the transcriptional regulatory role of FoxP1 on Arih2 via transcription factor prediction and dual‐luciferase reporter analysis. FoxP1 has been implicated in cell proliferation and differentiation in various tissues.^38^, ^39^ A recent study has revealed that the ability of MyoD to promote myogenic differentiation is impaired by FoxP1 expression.^40^ In addition, FoxP1 is a crucial modulator of cancer‐induced skeletal muscle atrophy.^41^ We also found that the FoxP1–Arih2 axis was involved in the regulation of cell metabolism by PDHB.

In summary, this study highlights the role of PDHB in myogenic differentiation and describes a novel mechanism whereby PDHB regulates myogenic differentiation via the FoxP1–Arih2 axis. Our results may contribute to the development of new therapeutic strategies for the management of sarcopenia.

## Conflicts of interest

The authors declare no competing interests related to this study.

All the mouse experiments were performed in accordance with the local and national ethical regulations. The authors of this manuscript certify that they comply with the ethical guidelines for authorship and publishing of *the Journal of Cachexia, Sarcopenia and Muscle*.^42^

## Funding

This work was supported by the National Natural Science Foundation of China (82000808), Natural Science Foundation of Jiangsu Province (BK20221091) and Chinese Postdoctoral Science Foundation (2022M711369).

## Supporting information


**Figure S1.** PDHB is up‐regulated during cardiotoxin (CTX)‐induced muscle regeneration.
(A) The H&E staining of the TA muscle from the control and CTX‐treated mice 3, 7 and 15 days post‐injury. Magnification = 200×, Scale bar = 50 μm. The mRNA (B) and protein (C) levels of PDHB during muscle regeneration were evaluated via qRT‐PCR and western blotting, respectively. The relative band intensities on the western blots were normalized to the β‐actin levels and analyzed using the ImageJ software. Data were expressed as mean ± SD and analyzed using Student's *t*‐test or one‐way ANOVA.* *P* < 0.05, ** *P* < 0.01, *** *P* < 0.001 vs. the control group.

**Figure S2.** Experimental validation of other genes (*Dcn*, *Bnip3l*, and *Zfp36l2*) predicted to be related to the regulation of cellular catabolic processes.
qRT‐PCR was performed to measure the mRNA levels of *Dcn* (A), *Bnip3l* (C), and *Zfp36l2* (E) after each siRNA was transfected. Western blotting showed the protein levels of MyHC, MyoD, MyoG, and β‐actin in three groups (B), (D), and (F). Data were expressed as mean ± SD and analyzed using Student's *t*‐test or one‐way ANOVA.* *P* < 0.05, ** *P* < 0.01, *** *P* < 0.001.

**Figure S3.** Experimental validation of the transcriptional regulatory role of Stat5a on *Arih2*.
qRT‐PCR was performed to measure the mRNA levels of *Stat5a* (A) and *Arih2* (B). (C) Western blot showing the protein levels of MyHC, MyoD, MyoG, and β‐actin in four groups. Data were expressed as mean ± SD and analyzed using Student's *t*‐test or one‐way ANOVA.* *P* < 0.05, ** *P* < 0.01, *** *P* < 0.001.

**Figure S4.** PDHB‐Arih2‐FoxP1 axis regulates HSkMC myogenic differentiation and lactate production.
(A) The mRNA expression of FoxP1 in HSkMC. (B) qRT‐PCR was performed to measure the mRNA levels of Arih2 in four groups. (C) Western blot analysis of MyHC, MyoD, MyoG and β‐actin in four groups. The relative protein levels were quantified (right). (D) Lactic acid content in C2C12 cells (left) and HSkMC (right). Data were expressed as mean ± SD and analyzed using Student's *t*‐test or one‐way ANOVA.* *P* < 0.05, ** *P* < 0.01, *** *P* < 0.001.

**Figure S5.** PDHB overexpression promotes myogenic differentiation in C2C12 cells.
C2C12 cells were transfected with pcDNA3.1 or pcDNA3.1‐PDHB and then induced to differentiate in the DM for 3 d. (A) qRT‐PCR (left) and western blotting (right) were performed to detect PDHB mRNA and protein levels. (B) The expressions of *Myf5*, *MyoD*, *MyoG*, and *MyHC* mRNA were calculated by qRT‐PCR analysis. (C) Western blotting showing the protein levels of MyHC, MyoD, MyoG, and β‐actin. The relative levels of the target proteins were normalized to those of β‐actin. (D) MyHC (green) immunofluorescence staining was used to assess the myotube formation of C2C12 cells transfected with pcDNA3.1 or pcDNA3.1‐PDHB. The cell nucleus was stained with DAPI (blue). Scale bar = 100 μm. The fusion index (the percentage of nuclei in fused myotubes out of the total nuclei) was calculated. Data are shown as mean ± SD. * *P* < 0.05, ** *P* < 0.01, *** *P* < 0.001 (Student's *t*‐test) vs. the pcDNA3.1 group.

**Table S1.** The sequences of siRNAs used for RNA interference (The prefix letter m for mice and h for human)

**Table S2.** Primer sequences used for qRT‐PCR (The prefix letter m for mice and h for human)
