# Supplementary figures and images for: Pyruvate dehydrogenase B regulates myogenic differentiation via the FoxP1–Arih2 axis
Source: J Cachexia Sarcopenia Muscle. 2022 Dec 23;14(1):606–21. doi: 10.1002/jcsm.13166 (PMC9891931; doi:10.1002/jcsm.13166)

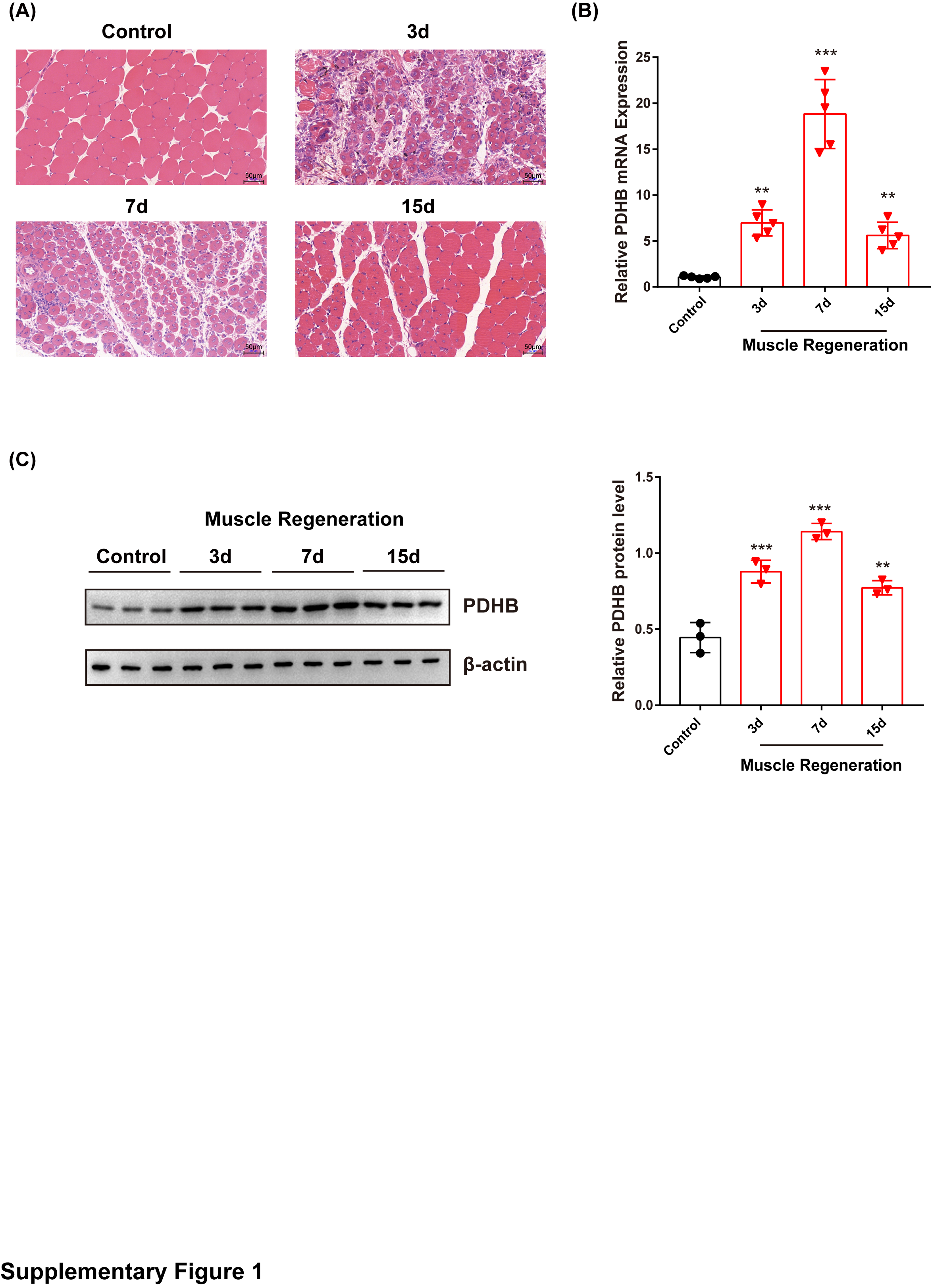

Supplement: Supplementary file 1 — Figure S1. PDHB is up‐regulated during cardiotoxin (CTX)‐induced muscle regeneration. (A) The H&E staining of the TA muscle from the control and CTX‐treated mice 3, 7 and 15 days post‐injury. Magnification = 200×, Scale bar = 50 μm. The mRNA (B) and protein (C) levels of PDHB during muscle regeneration were evaluated via qRT‐PCR and western blotting, respectively. The relative band intensities on the western blots were normalized to the β‐actin levels and analyzed using the ImageJ software. Data were expressed as mean ± SD and analyzed using Student's t‐test or one‐way ANOVA.* P < 0.05, ** P < 0.01, *** P < 0.001 vs. the control group. [file JCSM-14-606-s003.tif]

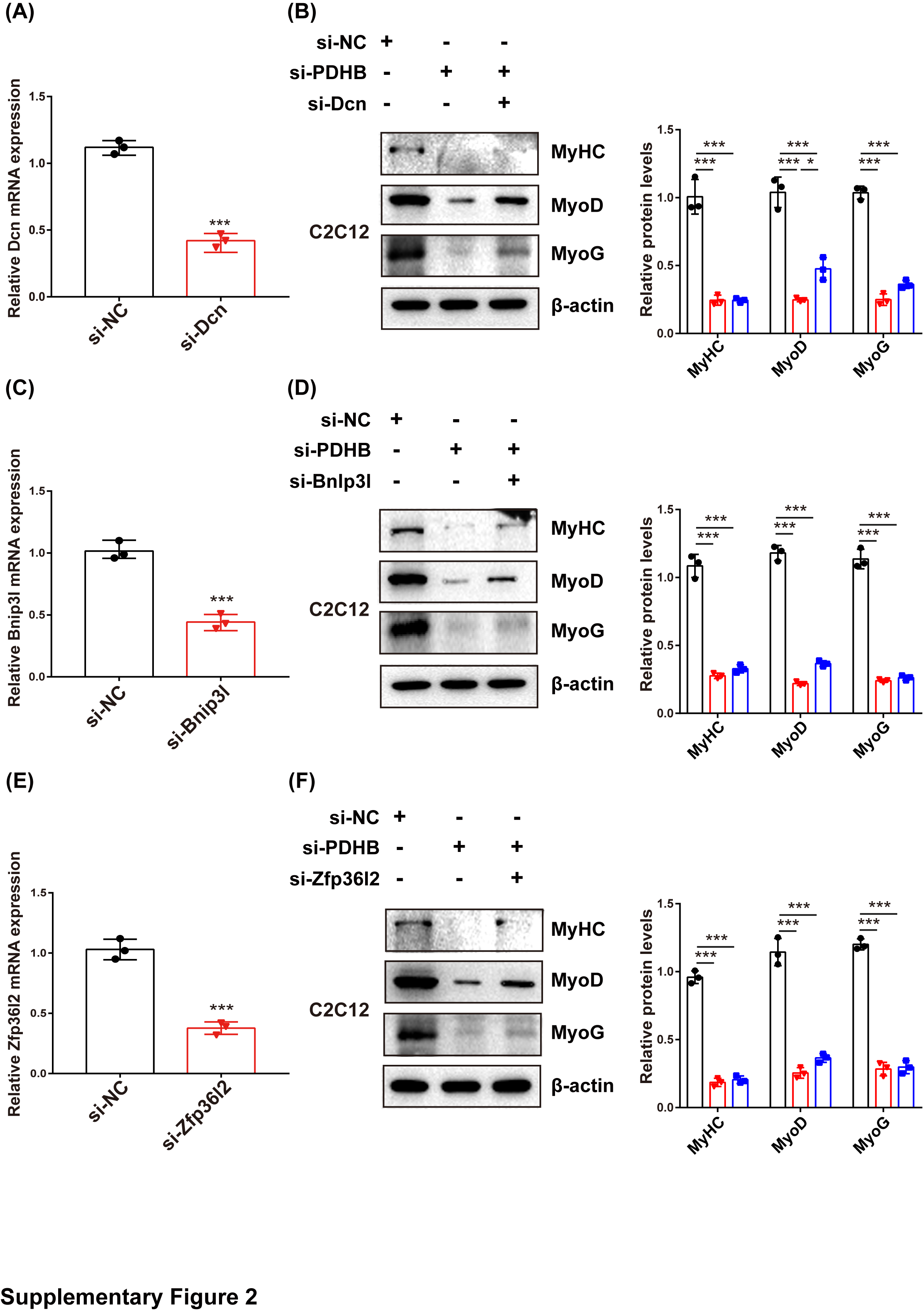

Supplement: Supplementary file 2 — Figure S2. Experimental validation of other genes (Dcn, Bnip3l, and Zfp36l2) predicted to be related to the regulation of cellular catabolic processes. qRT‐PCR was performed to measure the mRNA levels of Dcn (A), Bnip3l (C), and Zfp36l2 (E) after each siRNA was transfected. Western blotting showed the protein levels of MyHC, MyoD, MyoG, and β‐actin in three groups (B), (D), and (F). Data were expressed as mean ± SD and analyzed using Student's t‐test or one‐way ANOVA.* P < 0.05, ** P < 0.01, *** P < 0.001. [file JCSM-14-606-s006.tif]

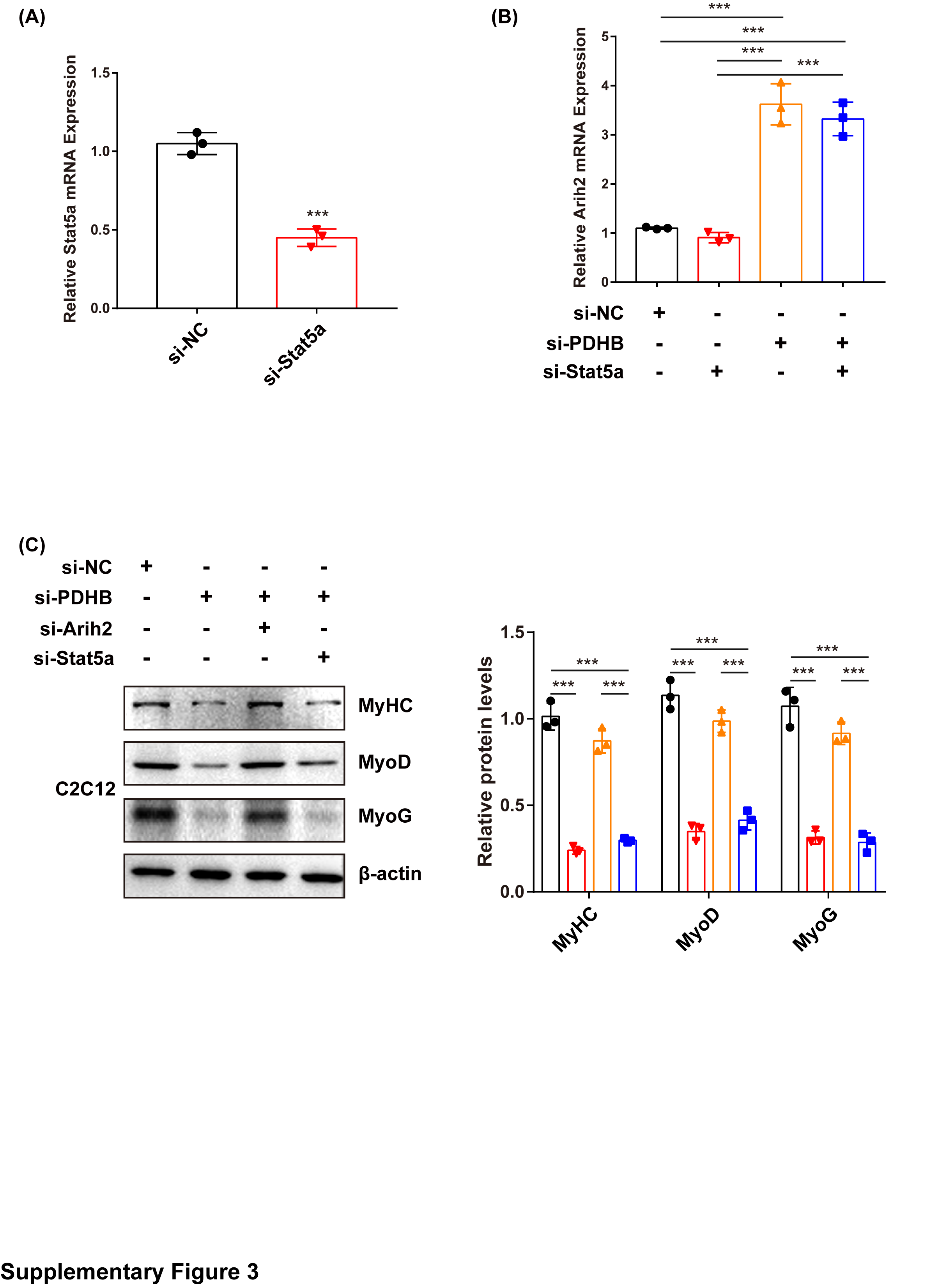

Supplement: Supplementary file 3 — Figure S3. Experimental validation of the transcriptional regulatory role of Stat5a on Arih2. qRT‐PCR was performed to measure the mRNA levels of Stat5a (A) and Arih2 (B). (C) Western blot showing the protein levels of MyHC, MyoD, MyoG, and β‐actin in four groups. Data were expressed as mean ± SD and analyzed using Student's t‐test or one‐way ANOVA.* P < 0.05, ** P < 0.01, *** P < 0.001. [file JCSM-14-606-s007.tif]

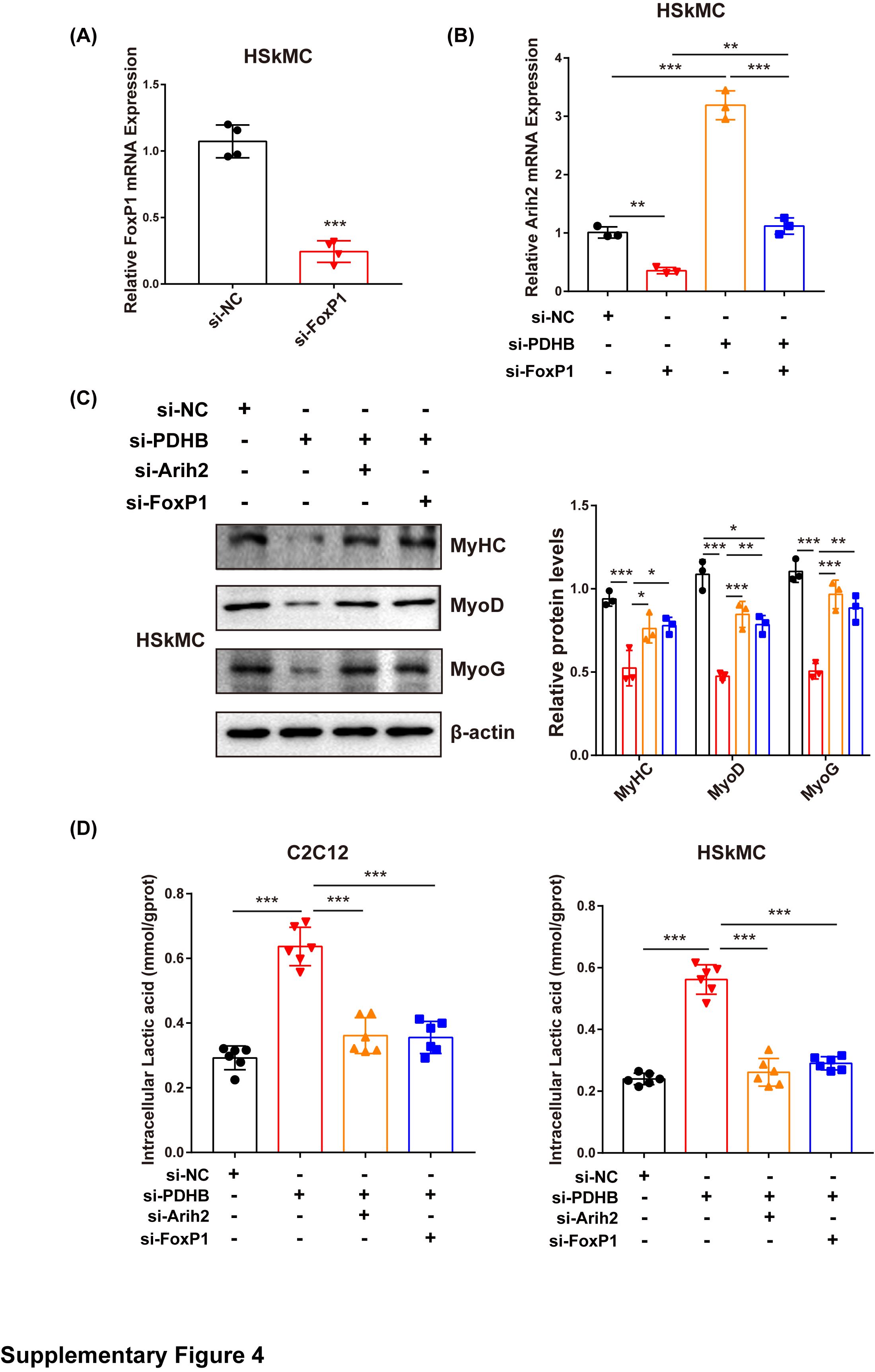

Supplement: Supplementary file 4 — Figure S4. PDHB‐Arih2‐FoxP1 axis regulates HSkMC myogenic differentiation and lactate production. (A) The mRNA expression of FoxP1 in HSkMC. (B) qRT‐PCR was performed to measure the mRNA levels of Arih2 in four groups. (C) Western blot analysis of MyHC, MyoD, MyoG and β‐actin in four groups. The relative protein levels were quantified (right). (D) Lactic acid content in C2C12 cells (left) and HSkMC (right). Data were expressed as mean ± SD and analyzed using Student's t‐test or one‐way ANOVA.* P < 0.05, ** P < 0.01, *** P < 0.001. [file JCSM-14-606-s002.tif]

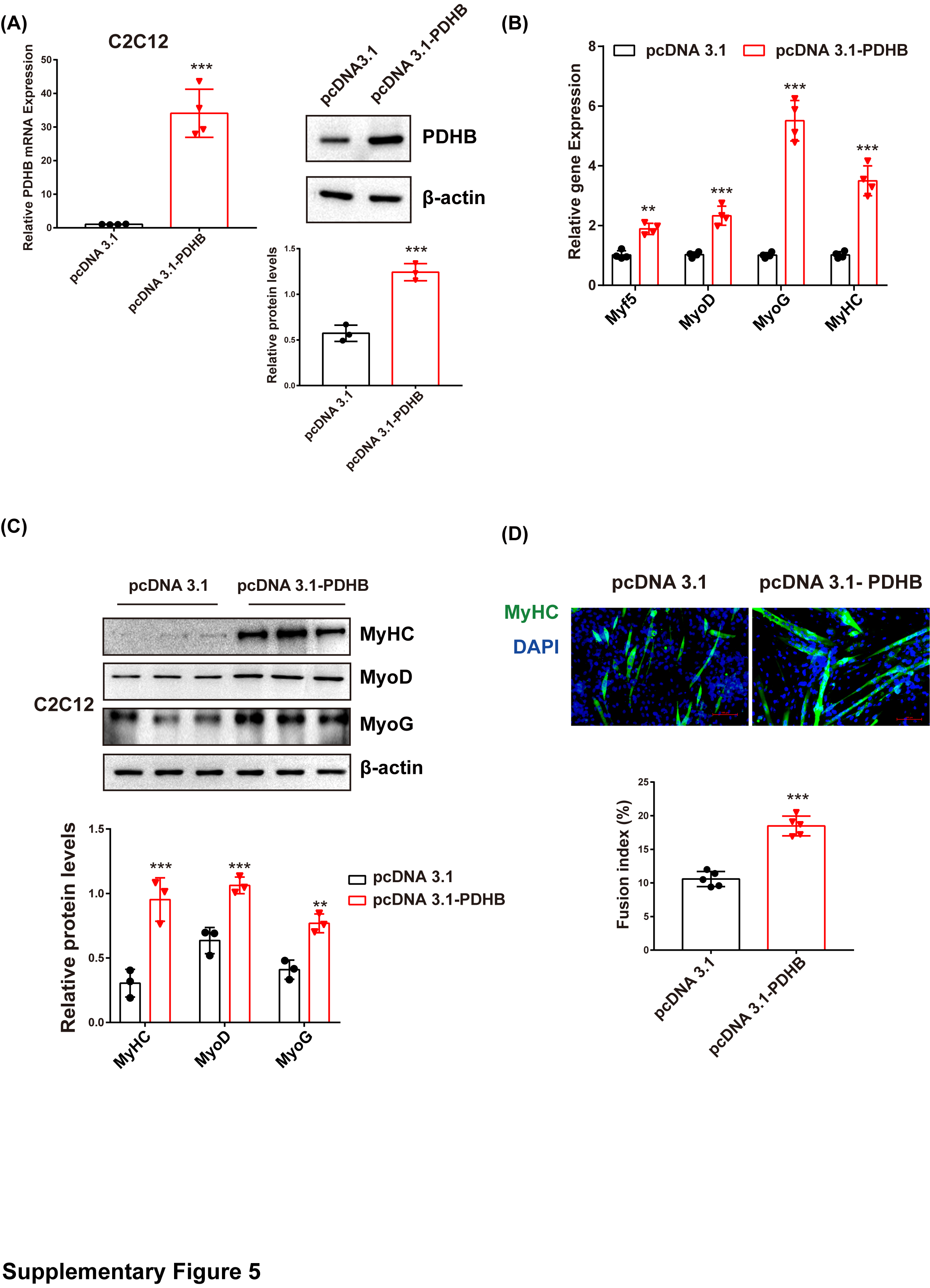

Supplement: Supplementary file 5 — Figure S5. PDHB overexpression promotes myogenic differentiation in C2C12 cells. C2C12 cells were transfected with pcDNA3.1 or pcDNA3.1‐PDHB and then induced to differentiate in the DM for 3 d. (A) qRT‐PCR (left) and western blotting (right) were performed to detect PDHB mRNA and protein levels. (B) The expressions of Myf5, MyoD, MyoG, and MyHC mRNA were calculated by qRT‐PCR analysis. (C) Western blotting showing the protein levels of MyHC, MyoD, MyoG, and β‐actin. The relative levels of the target proteins were normalized to those of β‐actin. (D) MyHC (green) immunofluorescence staining was used to assess the myotube formation of C2C12 cells transfected with pcDNA3.1 or pcDNA3.1‐PDHB. The cell nucleus was stained with DAPI (blue). Scale bar = 100 μm. The fusion index (the percentage of nuclei in fused myotubes out of the total nuclei) was calculated. Data are shown as mean ± SD. * P < 0.05, ** P < 0.01, *** P < 0.001 (Student's t‐test) vs. the pcDNA3.1 group. [file JCSM-14-606-s001.tif]
