# Supplementary material for: Pyruvate dehydrogenase B regulates myogenic differentiation via the FoxP1–Arih2 axis
Source: J Cachexia Sarcopenia Muscle. 2022 Dec 23;14(1):606–21. doi: 10.1002/jcsm.13166 (PMC9891931; doi:10.1002/jcsm.13166)
Supplement: Supplementary file 6 — Table S1. The sequences of siRNAs used for RNA interference (The prefix letter m for mice and h for human) [file JCSM-14-606-s004.docx]

**­­Supplementary materials**

**Table S1. The sequences of siRNAs used for RNA interference (The prefix letter m for mice and h for human)**

| **Target** | **Sense (5’→ 3’)** | **Antisense (5’→ 3’)** |
| --- | --- | --- |
| m-PDHB | CCUGCAUUCAACUUCCUUGAUTT | AUCAAGGAAGUUGAAUGCAGGTT |
| m-Arih2 | CGCUACCUCUUUAGGGACUAUTT | AUAGUCCCUAAAGAGGUAGCGTT |
| m-Zfp36l2 | CAAACCUCAAUCUGAACAACATT | UGUUGUUCAGAUUGAGGUUUGTT |
| m-Bnip3l | GCAAUGGCAAUGAGAAUGGAATT | UUCCAUUCUCAUUGCCAUUGCTT |
| m-Dcn | CCUGAAGGACUUGCAUACCUUTT | AAGGUAUGCAAGUCCUUCAGGTT |
| m-Stat5a | GACCAAGAUGGCGAGUUUGTT | CAAACUCGCCAUCUUGGUCTT |
| m-FoxP1 | GUGCGAGUAGAGAACGUUAAATT | UUUAACGUUCUCUACUCGCACTT |
| h-PDHB | GACAGUUCGUGAUGCUAUAAATT | UUUAUAGCAUCACGAACUGUCTT |
| h-Arih2 | GCUGGAUGUGUCUAGGAGAUUTT | AAUCUCCUAGACACAUCCAGCTT |
| h-FoxP1 | GCAGCAAGUUAGUGGAUUAAATT | UUUAAUCCACUAACUUGCUGCTT |
