# Supplementary material for: Pyruvate dehydrogenase B regulates myogenic differentiation via the FoxP1–Arih2 axis
Source: J Cachexia Sarcopenia Muscle. 2022 Dec 23;14(1):606–21. doi: 10.1002/jcsm.13166 (PMC9891931; doi:10.1002/jcsm.13166)
Supplement: Supplementary file 7 — Table S2. Primer sequences used for qRT‐PCR (The prefix letter m for mice and h for human) [file JCSM-14-606-s005.docx]

**­­Supplementary materials**

**Table S2. Primer sequences used for qRT-PCR (The prefix letter m for mice and h for human)**

| **Gene** | **Forward Primer (5’→ 3’)** | **Reverse Primer (5’→ 3’)** |
| --- | --- | --- |
| m-PDHB | CGGTGCAGTTGACAGTTCGT | TCTTCCCCAAGCAGAAAAACTTT |
| m-MyoD | ATGATGACCCGTGTTTCGACT | CACCGCAGTAGGGAAGTGT |
| m-MyoG | GCAGGCTCAAGAAAGTGAATGA | TAGGCGCTCAATGTACTGGAT |
| m-MyHC | CTCTTCCCGCTTTGGTAAGTT | CAGGAGCATTTCGATTAGATCCG |
| m-Arih2 | GAAGAGGACTATGACCCAAACTG | CTTTAAGACAGAGGCTAAGCTGG |
| m-Mettl16 | GACAAACCACCTGACTTCGCA | TCTGACTGCTTCGGGGTCTT |
| m-Npm1 | ATGGAAGACTCGATGGATATGGA | ACCGTTCTTAATGACAACTGGTG |
| m-Abhd5 | TGGTGTCCCACATCTACATCA | CAGCGTCCATATTCTGTTTCCA |
| m-Zfp36l2 | AGCGGCTCCCAGATCAACT | ACTTCTCGCCGTACTTGCAC |
| m-Ubxn1 | TCGAGGCTGCGATGGATTG | CAGGGCCAACTTGCTCTGAG |
| m-Bnip3l | CTGGAGCACGTTCCTTCCTC | ACAGTGCGAACTGCCTCTTG |
| m-Cav1 | ATGTCTGGGGGCAAATACGTG | CGCGTCATACACTTGCTTCT |
| m-Dcn | TCTTGGGCTGGACCATTTGAA | CATCGGTAGGGGCACATAGA |
| m-Bcap31 | GCCACCTTCCTCTACGCAG | TGCCATAGGTCACTACCAACTC |
| m-Clec16a | GCAAGTCCTCCCGCAACAT | GAGCAGGTTCCGATTTTGTTCT |
| m-Snx9 | ACCAAGGCTCGGGTCATGTA | ACCAACATTCGGGTTTGTAACTG |
| m-Atpif1 | GGTGTCTGGGGTATGAAGGTC | CCTTTTCTCGTTTTCCGAAGGC |
| m-N4bp1 | TTACGTTTCTACTGCACTTAGCG | AGAGGGTCATCAGGAACCATAA |
| m-Stat5a | CAGCCGTGGGATGCTATTGA | GGGACAGCGGTCATACGTG |
| m-FoxP1 | CACCTCAGGTTATCACTCCTCA | AGCTGCAACTGTTCCTGTTGT |
| m-β-actin | GTGACGTTGACATCCGTAAAGA | GCCGGACTCATCGTACTCC |
| h-PDHB | AAGAGGCGCTTTCACTGGACA | ACTAACCTTGTATGCCCCATCA |
| h-Myf5 | AACCCTCAAGAGGTGTACCAC | AGGACTGTTACATTCGGGCAT |
| h-MyoD | CGGCATGATGGACTACAGCG | CAGGCAGTCTAGGCTCGAC |
| h-MyoG | GGGGAAAACTACCTGCCTGTC | AGGCGCTCGATGTACTGGATA |
| h-MyHC | GGGAGACCTAAAATTGGCTCAA | TTGCAGACCGCTCATTTCAAA |
| h-Arih2 | TCCCGAGGAGTACCAGTTCAC | GCAGTTGAGCAGAATTGGACTT |
| h-FoxP1 | ATGATGCAAGAATCTGGGACTG | AGCTGGTTGTTTGTCATTCCTC |
| h-β-actin | CATGTACGTTGCTATCCAGGC | CTCCTTAATGTCACGCACGAT |
